# Development of a booster intervention for graded sensorimotor retraining (RESOLVE) in people with persistent low back pain: A nested, randomised, feasibility trial

**DOI:** 10.1002/msc.1715

**Published:** 2022-11-26

**Authors:** Edel T. O'Hagan, Aidan G. Cashin, Rodrigo R. N. Rizzo, Hayley B. Leake, Pauline Zahara, Matthew K. Bagg, Benedict M. Wand, James H. McAuley

**Affiliations:** ^1^ Centre for Pain IMPACT Neuroscience Research Australia Sydney New South Wales Australia; ^2^ Prince of Wales Clinical School University of New South Wales Sydney New South Wales Australia; ^3^ Westmead Applied Research Centre The University of Sydney Sydney New South Wales Australia; ^4^ School of Health Sciences Faculty of Medicine and Health University of New South Wales Sydney New South Wales Australia; ^5^ IIMPACT in Health Allied Health and Human Performance University of South Australia Adelaide South Australia Australia; ^6^ Curtin Health Innovation Research Institute Faculty of Health Sciences Curtin University Perth Western Australia Australia; ^7^ Perron Institute for Neurological and Translational Science Perth Western Australia Australia; ^8^ Faculty of Medicine, Nursing and Midwifery and Health Sciences The University of Notre Dame Australia Fremantle Western Australia Australia

**Keywords:** back care, booster, complex interventions, low back pain, self‐management

## Abstract

**Introduction:**

Low back pain contributes to an increasing global health burden exacerbated by unsustained improvements from current treatments. There is a need to develop, and test interventions to maintain initial improvements from low back pain treatments. One option is to implement a booster intervention. This study aimed to develop and test the feasibility of implementing a booster intervention delivered remotely to supplement the benefits from a complex intervention for chronic low back pain.

**Method:**

This study was nested in the RESOLVE trial. The booster intervention was developed by an expert group, including a clinical psychologist, exercise physiologist and physiotherapists, and based on a motivational interviewing framework. We developed a conversational flow chart to support the clinician to guide participants towards achieving their pre‐specified personal goals and future low back pain self‐management. Participants with chronic low back pain who were aged over 18 years and fluent in English were recruited. The booster intervention was delivered in one session, remotely, by telephone. The intervention was considered feasible if: participants were able to be contacted or <3 contacts were necessary to arrange the booster session; there were sufficient willing participants (<15% of sample unwilling to participate); and participants and research clinicians reported a perceived benefit of >7/10.

**Results:**

Fifty participants with chronic non‐specific low back pain were recruited to test the feasibility of implementing the booster intervention. Less than three contact attempts were necessary to arrange the booster session, only one participant declined to participate. Participants perceived the session to be beneficial; on a 0 to 10 scale of perceived benefit, the average score recorded was 8.3 (SD 2.0). Clinicians also reported a moderate perceived benefit to the participant; the average score recorded by clinicians was 6.3 (SD 1.6).

**Conclusion:**

We developed a step by step, simple booster intervention that was perceived to be beneficial to participants with chronic low back pain. The booster can feasibly be delivered remotely following a complex intervention.

## 1 INTRODUCTION

Chronic low back pain, that is, low back pain that has persisted for longer than 3 months (Koes et al., 2006), is a growing health problem (Abajobir et al., 2017). Between 1990 and 2019, low back pain caused one of the largest absolute increases in the number of days lost to disability of any health condition (Vos et al., 2020). A factor contributing to the burden of low back pain is that improvements in symptoms provided by effective treatments are not sustained over time (Bagg et al., 2017). Symptom reoccurrence is common, and people with low back pain often continue to seek healthcare (Da Silva et al., 2017). There is a need to develop and test interventions that can help sustain the initial improvements provided by effective treatments for low back pain.

Booster interventions are a strategy intended to sustain the initial effects of interventions. For example, additional booster doses are intended to sustain the initial immunological effects of vaccines. Similarly, booster sessions are intended to prolong the effects of cognitive behavioural therapy for binge‐eating (Schlup et al., 2009) and problem gambling (Hodgins et al., 2009). Booster interventions vary in the mode of delivery and intensity. Booster interventions may be delivered in person (Bennell et al., 2014; Nicolson et al., 2017), by text message (Suffoletto et al., 2016), the internet (Andersson et al., 2018), or through telehealth (Fleig et al., 2013; Goodie et al., 2020); either once, or repeatedly (Andersson et al., 2018; Bennell et al., 2014; Suffoletto et al., 2016). There is no specific guidance on what should comprise a booster intervention.

That said, booster interventions may be particularly helpful to sustain the effects of complex interventions (Schlup et al., 2009). Complex interventions are challenging to implement because they have multiple interacting components that target multiple mechanisms, requiring individualisation and follow‐up (Moore et al., 2015). Several complex interventions are recommended for managing chronic low back pain (Gutknecht et al., 2015; Miller et al., 2015; O’Keeffe et al., 2015; Wälti et al., 2015), for which booster interventions have been developed in some cases (Nicolson et al., 2017; Peterson, 2018; Schmidt et al., 2021). In this context, a booster intervention may facilitate the necessary individual follow‐up to maintain effects on challenging intervention targets, for example, beliefs or knowledge.

Graded sensorimotor retraining (RESOLVE) is a novel complex intervention for people with chronic low back pain that appears effective at decreasing pain intensity after 18 and 26 weeks, compared to sham (Bagg et al., 2022). RESOLVE is delivered in 12 sessions, approximately weekly, with intended ongoing effects. Several targets of intervention with RESOLVE are challenging; for example, addressing individual belief and knowledge structures, and restoring precision of sensorimotor neuraxes through dedicated retraining (Bagg et al., 2022; Wand et al., 2022). It is possible that a booster session of the RESOLVE intervention might contribute to the intended ongoing effects. The aims of the present study were to develop and test the feasibility of implementing a booster intervention delivered by telephone to supplement the RESOLVE intervention.

## 2 METHODS

### 2.1 Objectives

- Develop a single‐session telephone booster intervention to supplement a complex intervention for chronic low back pain.
- Test the feasibility of implementing the booster intervention

### 2.2 Study design

We conducted a prospective, two‐group, parallel, 1:1 randomised, feasibility trial. This study had ethics approval from the our institutions Human Research Ethics Committee (HC15357), was registered on the Australia New Zealand Clinical Trials Registry (Registration number 12619000749101) and followed a planned protocol (O’Hagan et al., 2019). The study is reported here following the CONSORT extension for randomised pilot and feasibility trials (Eldridge et al., 2016; Thabane et al., 2016) and the TIDieR checklist (Hoffmann et al., 2016).

### 2.3 Participants and procedures

This study was nested within a larger trial of a novel complex intervention called graded sensorimotor retraining (RESOLVE) (Bagg et al., 2022). Briefly, the RESOLVE trial was a two‐group, parallel, randomised controlled trial of 276 adults with chronic non‐specific low back pain. Participants were allocated 1:1 to either RESOLVE or a sham procedures and attention control intervention. RESOLVE included multiple components: pain neuroscience education; graded sensory training; graded motor imagery training; graded, precision‐focused, feedback‐enriched functional movement training; and goal setting (Wand et al., 2022).

Participants were recruited in Sydney, Australia from 2015 through 2019. Outcome data were collected at baseline and 18, 26 and 52‐weeks post‐allocation. The baseline (week 0) for this study was 18‐weeks post‐allocation in the RESOLVE trial. At this point, the RESOLVE trial interventions were complete, and the primary endpoint reached. The booster intervention was scheduled before the 52‐week follow up time point. Optional participation in this study was offered to all participants that consented to inclusion in the RESOLVE trial. People were included if they consented to this study and were subsequently allocated to the RESOLVE intervention group. Consenting participants allocated to the sham intervention in the RESOLVE trial were not included in this feasibility study. We recruited 50 participants with chronic non‐specific low back pain who had been included in the RESOLVE trial.

### 2.4 Allocation and masking

Participants were allocated (1:1) to receive either the booster intervention or the control intervention. The allocation sequence was generated by an independent researcher, using computer‐generated random numbers in blocks of 2, 4 and 6. The allocations were sealed in opaque envelopes. Subsequent to allocation, participants and clinicians were no longer masked to allocation group. An unblinded research assistant collected the procedure‐specific results. The participant‐specific results were analysed by a researcher blinded to group allocation. To minimise detection bias, an independent research assistant collected these data.

### 2.5 Study interventions

#### 2.5.1 Booster intervention

Participants allocated to the booster intervention group received the booster intervention in addition to the usual RESOLVE trial procedures. The booster intervention was a conversation between the participant and clinician delivered via the telephone in one session to facilitate the participant's attainment of their pre‐specified personal goal and future self‐management. The research clinician followed a flow diagram to direct the telephone conversation with the participant. The booster session was intended to accompany the RESOLVE trial, no new information or education was presented during the session. Where possible, the same researcher who delivered the RESOLVE intervention delivered the booster session to that participant. A brief description of the components of the booster intervention is provided below (and more detail in accordance with the TIDieR checklist in Appendix 1).

### 2.6 Reorientation to goals

To being, the research clinician asked the participant to quantify their progress toward a personal functional goal that they had identified during the RESOLVE trial. Participants rated their progress on a 0 to 10 scale, with a range from 0 meaning have not achieved their goal, to 10 meaning the goal was achieved. If the participant reported a score of 10, the clinical did not proceed with the booster session.

### 2.7 Motivational interviewing to support goal attainment

If the participant did not achieve their goal (reported a 9 or less on the 0 to 10 scale), they were asked to quantify their confidence and motivation to achieve their goal in the next year. The participant was asked to quantify their confidence on a 0 to 10 scale (range 0 [not at all confident] to 10 [very confident]). How important the goal was to the participant was measured by asking participants to rate their motivation on a 0 to 10 scale (range 0 [not at all important] to 10 [very important]) to achieve that goal. The research clinician based the decision to proceed with the booster session on the participants score, scores of seven or more on both scales were considered sufficient confidence and motivation to achieve their goal. If the participant scored less than seven on either scale, the research clinician proceeded with the booster intervention. The research clinician led a conversation using motivational interviewing techniques to increase the participants motivation and reduce their ambivalence towards achieving their goal. The clinician focused on reiterating key skills in the RESOLVE program following a DEARS motivational interviewing approach (Mullins‐Sweatt et al., 2020) (outlined in Appendix 2).

### 2.8 Final summary

To conclude, the research clinician reiterated the skills and knowledge learnt during the RESOLVE intervention and reflected participants' statements that emerged from the booster session to reinforce future self‐management.

#### 2.8.1 Booster intervention

The booster was designed to reinforce the training components of the RESOLVE intervention, with emphasis on facilitating the participant's attainment of their pre‐specified personal goal and future self‐management. The research team developed a theory‐ and evidence‐based booster intervention, based on a motivational interviewing framework (Medley & Powell, 2010). Motivational interviewing is an effective method of facilitating behaviour change, demonstrated in managing addiction and body weight (Frost et al., 2018). The booster intervention also drew on psychological theories of self‐determination and self‐efficacy (Smith et al., 2007). We enlisted an expert group (*n* = 4) to design a flow diagram to direct the telephone conversation between a research clinician and the participant (Appendix 3). The expert group consisted of three researcher clinicians with an interest in musculoskeletal health and one psychologist. Their research experience ranged from graduate to senior researchers, all clinicians were senior clinicians, each with over 15 years of clinical experience.

#### 2.8.2 Control intervention

Participants allocated to the control group of this study received no intervention. They continued their participation in the RESOLVE trial unchanged.

### 2.9 Data collection

Data were collected using secure electronic systems at baseline (prior to allocation), and at 8‐ and 34‐weeks post‐allocation. These time points were anchored to the RESOLVE trial as follows: baseline = 18‐weeks post‐allocation, 8‐weeks post‐allocation (feasibility study) = 26‐weeks post‐allocation (RESOLVE trial), and 34‐weeks post‐allocation (feasibility study) = 52‐weeks post‐allocation (RESOLVE trial). Baseline data on participant‐specific outcomes for the RESOLVE trial was used as baseline data for the Booster study and follow‐up data at 52 weeks post‐randomisation into the RESOLVE trial.

### 2.10 Outcomes

This feasibility study had no primary outcome. We measured both procedure‐ and participant‐specific outcomes. Novel feasibility thresholds for each procedure‐specific outcome were established.

#### 2.10.1 Procedure‐specific outcomes

These were (1) the number of efforts made to arrange the [booster intervention] telephone call (feasibility threshold: 2 or fewer efforts), (2) retraction of consent from a participant (feasibility threshold: >15% unwilling to participate (8 participants)), (3) the participants' perceived benefit of the intervention, and (4) the clinicians' perceived benefit. We developed two scales to understand the benefit of the booster session. Participants were asked to rate the benefit from the telephone call—towards helping achieve their back pain related goals—on an 11‐point numeric rating scale (range, 0 [not beneficial] to 10 [very beneficial]). Clinicians were asked to rate the benefit from the telephone call—towards helping achieve the participant's back pain related goals—on the same 11‐point numeric rating scale (range, 0 [not beneficial] to 10 [very beneficial]. The feasibility threshold on this scale is a median 8 or more points).

#### 2.10.2 Participant‐specific outcomes

These were pain intensity measured using an 11‐point numeric rating scale (Dworkin et al., 2015) (range, 0 [no pain] to 10 [worst pain imaginable pain]) and disability measured using the Roland Morris Disability Questionnaire (Roland & Morris, 1983) with a (range, 0–24 [with higher scores indicating greater levels of disability]).

### 2.11 Statistical analysis

We reported the mean and standard deviation (SD) for normally distributed variables, and the median and inter‐quartile range (IQR) for categorical or skewed variables. No comparison was performed for the procedure‐specific outcomes because these were only collected from the booster intervention group. Data on participant‐specific outcomes were analysed by intent‐to‐treat. We used independent *t*‐test to estimate the differences in means in participant outcomes between those who did and did not receive the booster intervention. We used *R*, version 3.4.2 (Team, 2012), to conduct the analysis.

## 3 RESULTS

Twenty‐five participants were randomly allocated to receive the booster intervention, and twenty‐five participants were assigned to the control group. Both groups were comparable concerning demographic characteristics at baseline. The group receiving the booster intervention reported higher levels of baseline depression and higher levels of pain catastrophising than the control group. All other clinical variables were similar across participants in both groups. Table 1 outlines the demographic and clinical characteristics of the participants.

**TABLE 1:** Demographic data

	Booster intervention *n* = 25	Control *n* = 25
Age in years, mean (SD)	43 (14.5)	50.6 (10.8)
Female, *n* (%)	16 (64%)	13 (52%)
Duration of low back pain in years, mean (SD)	7.6 (6.0)	7.8 (7.2)
First episode of low back pain, *n* (%)	11 (44%)	11 (44%)
Other pain areas, *n* (%)	13 (52%)	13 (52%)
Depressive symptoms (0–21), mean (SD)^a^	4 (4.6)	2.4 (2.8)
Pain catastrophising (0–52), mean (SD)^b^	19.9 (12.5)	14.2 (8.0)
Back beliefs (9–45), mean (SD)^c^	26.6 (6.0)	28.9 (6.5)
Kinesiophobia (17–68), mean (SD)^d^	41.0 (5.7)	39.2 (5.8)
Back self‐perception (0–36), mean (SD)^e^	5.8 (4.2)	6.0 (1.9)
Pain knowledge (0–19), mean (SD)^f^	4.3 (2.1)	5.8 (5.2)
Pain self‐efficacy (0–60), mean (SD)^g^	45.0 (11.7)	42.5 (11.0)
Sleep disturbance (0–28), mean (SD)^h^	12.2 (7.7)	14.7 (7.6)
Quality of life (5–25), mean (SD)^i^	10.0 (2.6)	9.9 (2.7)

^a^ Depression severity scale of Depression, Anxiety and Stress Scale with a range from 0 (no depressive symptoms) to 21 (high depressive symptoms).

^b^ Pain Catastrophising Scale with a range from 0 (no pain catastrophising) to 52 (elevated levels of pain catastrophising).

^c^ Back Beliefs Questionnaire with a range from 9 (less pessimistic beliefs) to 45 (more pessimistic beliefs).

^d^ Tampa Scale of Kinesiophobia with a range from 17 (less fearful of movement) to 68 (more fearful of movement).

^e^ Fremantle Back Awareness Questionnaire with a range from 0 (less severe levels of perceptual disruption) to 36 (more severe levels of perceptual disruption).

^f^ Neurophysiology of pain Questionnaire with a range from 0 (poor knowledge of the neurophysiology) of pain to 19 (good knowledge of the neurophysiology of pain).

^g^ Pain Self‐Efficacy Questionnaire with a range from 0 (low self‐efficacy) to 60 (high self‐efficacy).

^h^ Insomnia Severity Index with a range from 0 (low levels of insomnia) to 28 (elevated levels of insomnia).

^i^ European Quality of Life Five Dimension Five Levels questionnaire with a range from 5 (good health‐related quality of life) to 25 (poorer health‐related quality of life).

### 3.1 Procedure specific outcomes

#### 3.1.1 Number of contacts

Forty‐eight text messages were sent in total (median = 1, IQR 1–2). One participant preferred email contact and was emailed twice to organise the booster session. Twenty‐nine phone calls were conducted to arrange or reschedule the booster session (median = 1, IQR 0–1). One participant did not answer any phone calls; we interpreted this as withdrawal of consent.

#### 3.1.2 Duration of booster session

The total time spent for all booster sessions was 562 min (9 h 22 min), median 22 (IQR 19–29) minutes, per participant.

#### 3.1.3 Perceived benefit of booster session

On average, participants perceived the booster intervention as beneficial (mean 8.3 SD 2.0). Clinicians also perceived the session to be beneficial to the participant (mean 6.3 SD 1.6).

### 3.2 Participant specific outcomes

We collected participant specific data for pain intensity and disability. There was no difference in mean pain intensity at 52 weeks post‐randomisation in the participants who received a booster and the control group (Mean difference (MD) = −0.71, 95% confidence interval (CI) −1.99 to 0.95). There was no difference in disability at 52 weeks post‐randomisation in the participants who received a booster and the control group (MD = 0.11, 95% CI −2.37–2.64). There was no missing data.

### 3.3 Feasibility criteria

Less than three contact attempts were necessary to arrange the booster session, only one participant declined to participate. Feedback from participants suggested a perceived benefit above our feasibility threshold (7/10), but perceived benefit rated by research clinicians was lower than our feasibility threshold (6/10).

## 4 DISCUSSION

### 4.1 Main findings

We developed a theory‐ and evidence‐informed telephone delivered booster intervention to supplement a complex intervention for chronic low back pain. Based on a motivational interviewing framework, we used measures of confidence and importance as salient indicators of likely future goal achievement. Our results highlight that an additional 22 min of clinician time as a booster session delivered remotely was perceived as beneficial to participants and research clinicians in helping participants achieve their low back pain‐related goals. We achieved our pre‐determined threshold for perceived benefit rated by participants, but not our pre‐determined threshold for perceived benefit rated by research clinicians. Although the clinicians appeared to value the interaction less than participants the results suggest that this a promising intervention that should be tested in a large RCT to investigate the effect on participant outcomes.

The booster intervention drew on psychological theories of self‐determination and self‐efficacy and was developed using motivational interviewing principles. Motivational interviewing is a therapeutic framework to promote constructive engagement in clinical rehabilitation interventions (Medley & Powell, 2010). Critical components of motivational interviewing are its participant centred approach, upholding participant autonomy, commitment and readiness for change through empathic listening and collaborative decision‐making (Medley & Powell, 2010). Despite these components being encouraged for successful low back pain management, the use of motivational interviewing is in its infancy in low back pain (Holden et al., 2015). Clinicians are aware of the potential benefits of motivational interviewing; a survey of physiotherapists reported the importance of motivating individuals with low back pain to return to their usual activities (Holden et al., 2015). However, insufficient training or confidence with motivational techniques were cited as reasons why they do not use them (Holden et al., 2015). We provide a simple, step by step approach to motivational interviewing that could easily be employed by clinicians to facilitate goal attainment when used as a booster intervention for participants with chronic low back pain.

The majority of participants (23/25, 92%), rated the phone call as beneficial. One interpretation of this result is that it highlights the importance of validation for participants with chronic low back pain. A meta‐ethnography that synthesized 195 qualitative studies suggested that validation can empower a person with chronic pain to embark on a journey of recovery (Toye et al., 2020). Recently published research (O’Hagan et al., 2021) evaluated entries made by people with chronic low back pain on social media: content analysis of 768 posts indicated that almost half (49%) of original posts were consistent with seeking validation for their pain. The booster session interactions may have been beneficial at providing validation, and potentially have encouraged participants to independently undertake the steps towards goal achievement and increase their sense of mastery and ownership of their goal‐setting skills (Gardner et al., 2016). The difference in perceived benefit may reflect that communication is often undervalued by clinicians (Parker et al., 2020), but this booster trial highlights that participants valued the interaction. This trial was not powered to observe a difference between groups, however, the results suggest that the booster session is feasible and can be adapted for a large RCT.

### 4.2 Strengths and limitations

We prospectively registered the protocol for this study and reported the study following the CONSORT extension for randomised pilot and feasibility trials. We used a comprehensive and reproducible method to develop the booster intervention and created a template that could also provide guidance for future studies investigating the effect of booster sessions to extend the effect of complex interventions. We measured perceived benefit from the perspective of the clinician and the participant. Thus, we provide a balanced marker of satisfaction from both perspectives. Participant scores were collected by a research assistant to reduce the likelihood of a familiarity effect, whereby the participant rated the interaction highly due to liking the research clinician rather than the content of the interaction.

This study has some limitations. First, we acknowledge that there may be some responder bias because the included participants expressed an interest in health research and thus may not be wholly reflective of people with low back pain. Second, this trial was nested in the RESOLVE trial, restricting our sample size, and limiting our ability to investigate the effect on participant‐specific outcomes such as pain or disability. There may have been a ceiling effect of the interventions as both groups had a sustained effect from the RESOLVE intervention. In addition, we did not include consumers in the expert focus group, consumers may have provided valuable insights to help direct the booster phone call.

### 4.3 Clinical implications

Complex interventions for low back pain are more effective than simple interventions, but they are time‐consuming, and the benefits may diminish over time (Nijs et al., 2017). Long‐term management of low back pain should progress from receiving an intervention to self‐management. Preliminary qualitative results indicate that participants would like to receive further contact from clinicians, such as a telephone call, to reinforce the effect of complex interventions and facilitate ongoing self‐management. Recent evidence highlights that people value learning about pain (Leake et al., 2021) and those who understand the causes and consequences of low back pain are more likely to intend to self‐manage (O’Hagan et al., 2022). Effective self‐management requires the person to have, or develop, emotional, behavioural and technical skills (Corbin & Strauss, 1988). Motivational interviewing framework could be used by clinicians to identify those skills and support the transition to self‐management.

## 5 CONCLUSION

We outline a brief booster intervention to support the sustained benefits of a complex intervention. We developed a simple step by step, beneficial intervention that clinicians could feasibly implement into clinical practice with minimal additional cost.

## AUTHOR CONTRIBUTIONS

Edel T. O'Hagan conceived the study, provided methodological expertise, and wrote the protocol. Aidan G. Cashin provided methodological expertise. Rodrigo R. N. Rizzo provided methodological and clinical area expertise. Hayley B. Leake provided methodological expertise. Pauline Zahara provided methodological expertise. Matthew K. Bagg provided methodological expertise. Benedict M. Wand provided methodological expertise. James H. McAuley is the guarantor and conceived the study, provided clinical area expertise. All authors read, contributed to, and approved the final version of the manuscript.

## CONFLICT OF INTEREST

Matthew K. Bagg has received fees to speak about pain neuroscience and rehabilitation, and engagement with research evidence. The other authors have no interests to declare.

## ETHICS STATEMENT

The trial was approved by the University of New South Wales Human Research Ethics Committee (HC15357_11).

## Supporting information

Supporting Information S1

Supporting Information S2

Supporting Information S3

## Data Availability

The data that support the findings of this study are available from the corresponding author upon reasonable request.
