# Supplementary material for: Development of a booster intervention for graded sensorimotor retraining (RESOLVE) in people with persistent low back pain: A nested, randomised, feasibility trial
Source: Musculoskeletal Care. 2022 Nov 26;21(2):444–52. doi: 10.1002/msc.1715 (PMC10946532; doi:10.1002/msc.1715)
Supplement: Supplementary file 1 — Supporting Information S1 [file MSC-21-444-s002.docx]

Appendix 1- Description of the intervention (TIDieR checklist)

1. Name: Development of a booster intervention for graded sensorimotor retraining (RESOLVE) in people with persistent low back pain: a nested, randomised, feasibility trial.

2. Why: There are difficulties maintaining improvements in symptoms when achieved as part of a complex intervention, the RESOLVE trial tests one such intervention. The aim of a booster session is to investigate the feasibility of developing an intervention, remotely delivered, to maintain improvements in symptoms for people receiving a complex intervention. We expected the intervention to work by reinforcing the training components of the intervention. We investigated procedure specific outcomes to determine how acceptable adding a booster session to a trial with a complex intervention, such as RESOLVE is.

3. What (Procedures): Prospective, two-group 1:1 randomised, feasibility trial with blinding of assessors. Fifty participants were randomised to either receive a booster session or not. The booster session was delivered remotely in the form of a telephone call. As a feasibility study the primary outcome was not defined.

As this is the first trial the develop a booster intervention there is no exemplar. A key component of this feasibility study was to design a flow diagram to act as an exemplar (appendix 3). The flow diagram was designed by the research team with the guidance of a clinical psychologist.

Once the flow chart was finalised the researcher directed the phone call conversation in line with the flow chart. The researcher used individual functional goals that were outlined by the participant to direct the booster session. In our case the goals were identified in weeks 9-12 of the RESOLVE trial. The participant was asked to quantify their progress toward their functional goal by answering, “How close do you feel you are to achieving …. goal?” Participants will be asked to rate their answer on a 0-10 scale. If the participant reported that they had not achieved their goal the researcher used motivational interviewing techniques to facilitate the participant identifying their level of confidence and motivation toward achieving their goals. Although we expected this template to be applicable to other complex interventions for LBP, in this trial, participants were guided to existing trial resources introduced during the initial 12 interventions of the RESOLVE trial.

If the participant reported achieving their goals then the researcher reinforced their progress and encouraged them to set new goals.

This structure allowed the researcher to discuss progress toward goals, flare ups and future plans. It allowed time for reflection and reiteration of key components of the RESOLVE trial.

We investigated procedure specific outcomes. This included; number of contacts made to participant to arrange booster session, retraction of consent from participant, researcher time spent on phone call and perceived benefit of intervention.

We also investigated participant specific outcomes by investigating the difference from 18 weeks (primary outcome of RESOLVE trial) to 52 weeks, comparing those randomised to receive a booster session and those randomised to not receive a booster session over all measured outcomes.

We planned to collect data on patient specific outcomes including pain intensity, disability, depression, pain catastrophising, kinesiophobia, beliefs about back pain, pain self-efficacy, quality of life, healthcare resource use, and treatment credibility. After collecting the primary outcome RESOLVE trial funding was unavailable to collect data on all outcomes for all participants, instead a single additional observation was collected from participants who had not completed 52 week follow up. As a result we had data for analysis on pain intensity and disability only.

4. What (Materials): The booster session was scheduled between week 36 and week 42 post randomisation of the RESOLVE trial. Participants received one 30-60minute phone call to discuss their goals.

5. Who provided: Where possible the same researcher who delivered the RESOLVE intervention delivered the booster session to that participant. The researchers had one training session with a clinical psychologist to advise on how to conduct motivational interviewing over the telephone prior to the first booster session.

6. How: The participant was contacted in advance to arrange one 30-60minute phone call with the researcher. The initial contact was made via email by a research assistant who outlined the aim of the booster session. The researcher then contacted the participant at a specified time.

7. Where: The booster feasibility trial was conducted remotely. The researcher called the participant from the research lab at Neuroscience Research Australia at a time that wass convenient for the participant.

8. When and how much: The booster feasibility trial consisted of one 30-60minute phone call. Procedure specific outcomes were logged and tabulated. Outcome measures embedded in the RESOLVE trial served as participant specific outcomes.

9. Tailoring: The researcher tailored the booster session in line with individual goals that were pre-specified by the participant in weeks 9-12 of the RESOLVE trial.
