# Supplementary material for: Development of a booster intervention for graded sensorimotor retraining (RESOLVE) in people with persistent low back pain: A nested, randomised, feasibility trial
Source: Musculoskeletal Care. 2022 Nov 26;21(2):444–52. doi: 10.1002/msc.1715 (PMC10946532; doi:10.1002/msc.1715)
Supplement: Supplementary file 2 — Supporting Information S2 [file MSC-21-444-s001.docx]

### Appendix 2- DEARS approach used in the Booster session

|  | **Suggested statement or question** |
| --- | --- |
| ***D****evelop discrepancy* | It sounds like you have a lot going on, and these priorities are competing with your efforts to change at this time. |
|  | How do you think your life would be different if you achieved these goals? |
|  | What do you see your life like if you do not make changes? |
|  | What do you feel you need to change to obtain your goals? |
|  | How would you like things to be different? |
| ***E****xpress empathy* | I understand how difficult this is. |
|  | Yes, making changes is hard work…. It is very hard work. |
|  | I know where you are at with this. |
| ***A****mplifying Ambivalence* | How has your (e.g., pain) been a problem for you? How has it been a problem for others? |
|  | What was your life like before you had pain? |
|  | If you keep heading down the road you are on… what do you see happening?  That is ok if you do not want to work towards these goals, that is your choice. |
| ***R****olling with resistance* | Maybe you are not ready to change? |
|  | What do you want to do? How do you want to proceed? |
| ***S****tatements to support self-efficacy* | It seems as though you have put a lot of thought into your goals. |
|  | You have a good plan. |
|  | It sounds like you are still struggling with making these changes, but you have had some success at making some. |
|  | It sounds like you have made real progress. How does that make you feel? |
