# Supplementary material for: Development of a booster intervention for graded sensorimotor retraining (RESOLVE) in people with persistent low back pain: A nested, randomised, feasibility trial
Source: Musculoskeletal Care. 2022 Nov 26;21(2):444–52. doi: 10.1002/msc.1715 (PMC10946532; doi:10.1002/msc.1715)
Supplement: Supplementary file 3 — Supporting Information S3 [file MSC-21-444-s003.pdf]

Appendix 3- Flow chart of procedure for booster intervention

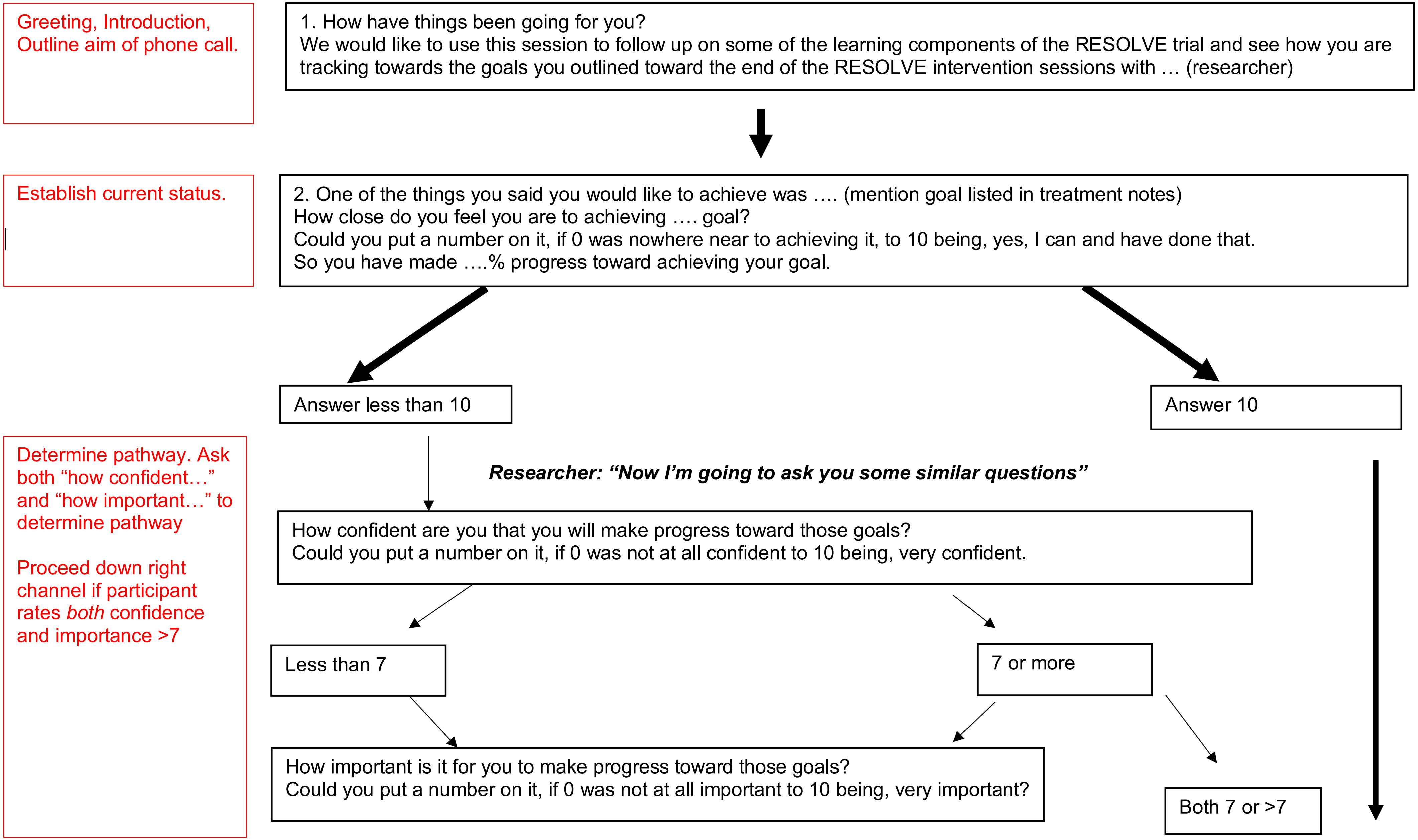

Explore motivation and importance, by following DEARS pathway.
If participant gets stuck choose some boxes on the right to remind the participant of components of trial and strategies used to target these components.

Less than 7

Researcher: "Let's explore that in a bit more detail"
"How about we consider some of the elements of the trial"

7 or more

***D**evelop discrepancy*
It sounds like you have a lot going on, and these priorities are competing with your efforts to change at this time.
How do you think your life would be different if you achieved these goals?
What do you see your life like if you don't make changes?
On the one hand you say that …. (e.g. going to the gym) is important to you, yet you continue to …. (e.g. put it off, prioritise other things), help me to understand…
What do you feel you need to change to obtain your goals?
You said you had made (e.g. …. progress), why not …?
-How would you like things to be different?
-If you do continue to work towards these goals how do you feel your life will be different from how it is today?

***E**xpress empathy*
I understand how difficult this is
Yes, making changes is hard work…. It is very hard work.
I know where you're at with this.
That must have been hard on you.

***A**mplifying Ambivalence*
How has your (e.g. pain) been a problem for you? How has it been a problem for others?
What was your life like before you had pain?
if you keep heading down the road you're on… what do you see happening?

Goal setting
-Can you tell me about your experience with goal setting?
-Can you tell me about that?

Tools for researcher-
Twin Peaks
Pacing
Hurt does not mean harm.
Motion is lotion.

Functional Movement training
-Do you remember any of the movement training that we did?
-Tell me about that?

Tools for researcher-
Forward-backward, side to side, rotation movements of Lx.
Flex/Ext, side flex, rotation movements of Lx.

(cont.)

***R**olling with resistance*
That is ok if you don't want to work towards these goals, that is your choice.
Maybe you aren't ready to change.
What do you want to do? How do you want to proceed?
Where do you want to go from here?

***S**upporting Self-efficacy*
It seems as though you have put a lot of thought into your goals?
You have a good plan?
It sounds like you are still struggling with making these changes, but you have had some success at making some.
It sounds like you have made real progress. How does that make you feel?
How do you feel about the progress you made?
-Can you remember anything that you found particularly useful when participating in the RESOLVE trial.
-Have you tried any of the strategies that you learned during the RESOLVE trial? (movement training, motor training, sensory re-training, challenging unhelpful beliefs)

Motor Training
-Do you remember watching the movement videos?
-Tell me about that?
-Why did we do that?

Tools for researcher-
Simple, complex, context videos.
Watch, visualise.
Pain relies on context

Sensory Training
-Do you remember identifying the dots or letters or numbers on your back?
-Tell me about that?
-Why did we do that?

Tools for researcher-
Two-point discrimination
Smudging

Pain education
-Do you remember what we talked about in the first 2 sessions?
-Tell me about that?
-Why did we do that?

Tools for researcher-
Pain is a product of the brain
The protective NS
Pain relies on context
Videos, EP

-Would you like to set new additional goals?
-What would they be?
-How do you plan on achieving them?
- Do you remember any of the reading/ sensory/ movement training that we did?
-Can you tell me about that?

Reflect on learning outcomes achieved and learning outcomes to review
i.e healthy mind, healthy body, healthy brain.

Let me see if I have understood everything so far….
(Give attention to progress made)
Did I miss anything?
Do you want to add anything or correct anything?
Can you tell me about that?
How confident do you feel moving forward?
How important is it for you to move forward?
Do you think it would be beneficial to revisit some of the elements of the trial again? Option for researcher to loop back to ***Researcher: "How about we consider some of the elements of the trial".***
Is there anything else you would like to discuss today?

Instil confidence, encouragement and wrap up

I appreciate you taking time to talk to me today
You are clearly a very resourceful person
You handled yourself really well in …. situation
That's a good suggestion
If I were in your shoes I don't know if I could have managed nearly so well
I've enjoyed talking with you today
